# Efficiency under epistasis of haplotype-based genomic selection for pure line breeding

**DOI:** 10.1038/s41437-026-00865-2

**Published:** 2026-07-11

**Authors:** Jean Paulo Aparecido Silva, José Marcelo Soriano Viana

**Affiliations:** Department of General Biology, Federal University of Viçosa, Viçosa, MG Brazil

**Keywords:** Plant breeding, Plant breeding

## Abstract

Haplotype-based genomic selection is considered a more efficient alternative for genomic selection under epistasis. We evaluated the efficacy of haplotype-based genomic selection under seven digenic epistasis types and the significance of including the additive x additive (AxA) effect in selection in pure line breeding. We simulated a genome composed of 10 chromosomes of 100 cM, including 1000 genes and 49,825 SNPs. We assumed positive dominance and 30% epistatic genes. We performed genomic selection using haplotypes of sizes 3, 6, and 9. We also processed low-cost genomic selection using 20% of the SNPs. We assessed selection efficacy using realized total genetic gain. We also evaluated the correlation between prediction accuracy and realized genetic gain and the decrease in genotypic variance over generations. Regardless of the epistasis type and SNP density, genomic selection was an efficient procedure for the development of superior pure lines. There was no effect of haplotype size on the efficacy of genomic selection. Under no selection, the AxA genetic value was negatively correlated with low magnitude ( < −0.25) with the number of favorable genes. Under selection, the inclusion of the predicted AxA value for selection did not increase the selection efficacy. Haplotype- and SNP-based genomic selection processes were equally efficient. Compared with traits determined only by additive and dominance gene effects, epistasis can decrease the total genetic gain. Under epistasis, there was a highly positive correlation between genetic gain and prediction accuracy. There was no difference between genomic selection procedures in terms of the decrease in genotypic variance.

## Introduction

Genomic selection has been successfully employed for the improvement of self-pollinated crops such as soybean, wheat, and rice, mainly through single nucleotide polymorphism (SNP)-based approach (Baertschi et al. 2021; Bandillo et al. 2023; Tessema et al. 2020). Alternatively, breeders can use haplotype-based genomic prediction. A haplotype is a group of linked genes and/or molecular markers along a chromosome which tend to be inherited together. This means that specifying a haplotype requires a criterion. The most used criterion was proposed by Gabriel et al. (2002). Breeders regularly define haplotypes based on a fixed or variable number of SNPs or length (kb) (Bhat et al. 2021). The referred advantages of the haplotype-based approach include that haplotypes can better capture and preserve linkage disequilibrium (LD) and genomic similarity for many generations (Bhat et al. 2021), that haplotypes are in stronger LD with quantitative trait loci (QTLs) than are individual SNPs (Cuyabano et al. 2014), and that haplotypes can better capture causal variant effects, IBD alleles, recent relationships, and local epistasis (Karimi et al. 2018; Teissier et al. 2020; Weber et al. 2023).

Weber et al. (2023) evaluated the accuracy of haplotype-based genomic prediction and reported inconsistent results across traits, species, and models. Processing a wheat dataset, greater increases in prediction accuracy from haplotyping, compared to the SNP-based approach, were observed for grain yield and sedimentation value. The accuracies increased from 0.70 to 0.82, from 0.49 to 0.62, and from 0.49 to 0.64, depending on the method of haplotyping and the statistical model used. Processing a soybean, maize, and canola datasets, no differences were observed between the haplotype- and SNP-based approaches. Sallam et al. (2020) observed more consistent benefits from haplotype-based genomic predictions. Their investigation evaluated haplotypes of varying sizes under two cross-validation schemes. All haplotype sizes outperformed the SNP-based approach for grain yield and protein content, with a haplotype size of 15 providing the greatest increase in prediction accuracy. In general, stratified sampling enhanced the predictive performance, achieving up to a 14.3% improvement for grain yield and a 16.8% improvement for protein content. These studies clearly demonstrate that although haplotype-based genomic prediction holds promise, its success is highly contingent upon multiple interacting factors, such as the model, trait architecture, species, population, haplotyping method, and training set.

In recent years, breeders have investigated the significance of fitting epistatic effects in genomic selection, with contrasting results. Weber et al. (2023) included additive x additive (AxA) effects into haplotype-based genomic prediction but observed only marginal improvements in prediction accuracy for most traits. For example, in wheat, grain yield prediction and its epistatic extension showed a negligible increase, from 0.80 to 0.81. In soybean, the same model improved the prediction accuracy for oil content from 0.68 to 0.69, with no increase for protein content. Miller et al. (2023) evaluated parent selection strategies in soybean breeding and reported that including AxA effects led to a greater than 50% increase in prediction accuracy, depending on the selection method. García-Barrios et al. (2024), who studied multienvironment genomic prediction in wheat, found no significant improvement in prediction accuracy by including AxA interactions compared with a purely additive model. Modeling epistasis in either whole-genome or subgenome models, Tessele et al. (2025) observed only a 3% improvement in genomic prediction accuracy.

Quantifying the impact of epistasis and haplotyping on selection has been a challenge in plant and animal breeding (Misztal et al. 2021). However, assessing the significance of epistasis in empirical datasets remains difficult because epistatic effects may have limited or nonsignificant contributions to genotypic value and genetic variance. To our knowledge, there is no simulation-based investigation on haplotype-based genomic selection under epistasis in pure line breeding. Notably, epistasis types were generally ignored in simulation-based studies on genomic selection (Nannuru et al. 2025; Seck et al. 2024). When epistasis was included, there was no specification of the epistasis type (Ali et al. 2020; Wientjes et al. 2022). In this simulation-based study, we evaluated the efficacy of haplotype-based genomic selection under seven digenic epistasis types, compared the efficacy assuming high- and low-SNP densities, and evaluated the importance of including the AxA effect in selection.

## Materials and methods

### Simulation

We used *REALbreeding* software (Viana et al. 2025) (proprietary; available upon request) to simulate a genome including 10 chromosomes of 100 cM, 1000 minor genes (100/chromosome), and 49,823 SNPs, comparable to the number of SNPs in the SoySNP50K chip. The number of SNPs/chromosome ranged from 4942 to 5000. The average densities for genes and SNPs were 1 and 0.02 cM, respectively. The simulated trait was grain yield in soybean, assuming a planting density of 300,000 plants per hectare. The software uses input from the user to simulate individual genotypes for genes and molecular markers and phenotypic values. The simulation process involves three stages: genome simulation, population simulation, and trait simulation. By using quantitative genetics theory, the software computes individual additive (A), dominance (D), and epistatic genetic values, general and specific combining ability effects, or genotypic values, depending on the population. The software also computes genetic variances and covariances considering the LD for genes and markers.

As inputs for the software, we defined, assuming no epistasis, the minimum genotypic and phenotypic values as 7 and 4 g/plant, respectively, with maximum values of 16 and 20 g/plant, respectively. The degree of dominance ranged from 0 to 1.2, with a mean of 0.6. The broad-sense heritability was set at 30% in each generation. We assumed digenic epistasis for 30% of the genes, resulting in 150 epistatic pairs. The following epistasis types were modeled: complementary (9:7 in F₂), duplicate (15:1 in F₂), dominant (12:3:1 in F₂), recessive (9:3:4 in F₂), dominant and recessive (13:3 in F₂), duplicate genes with cumulative effects (9:6:1 in F₂), and non-epistatic gene interaction (9:3:3:1 in F₂). We also assumed an admixture of types. For admixture, *REALbreeding* randomly assigns an epistasis type to each pair of interacting genes. For each epistasis type, we kept the same pairs of epistatic genes.

For each epistatic pair, the parametric values of the 36 parameters (population mean, four gene additive effects, six dominance effects, four AxA effects, six AxD effects, six DxA effects, and nine DxD effects) for the nine genotypic values were obtained by solving the equation system $\beta ={({X}^{{\prime} }{VX})}^{-1}X{\prime} {Vy}$ under the restrictions defined by Kempthorne (1954). β is the parameter vector, X is the incidence matrix, $V={diagonal}\left\{{f}_{{ij}}^{n}\right\}$ is the matrix of the genotype probabilities, n is the generation, and y is the vector of the genotypic values (${G}_{{ij}}$) (i, j = 0, 1, and 2). See details on Viana and Garcia (2022).

### Simulated scenarios

The base population consisted of 250 F₂ individuals derived from a cross between two contrasting pure lines, one carrying 70% and the other 30% of the favorable alleles. From the F_3_ to F_8_ generations, we performed haplotype-based genomic selection based on the predicted sum of the A and AxA values, using haplotype sizes of 3, 6, and 9 adjacent SNPs (H3, H6, and H9,A + AA, respectively). These numbers of SNPs represent the fixed number of adjacent SNPs employed in empirical- and simulation-based studies, although the upper limits have previously been set as 10, 20, 40, 50, and 100 (Calus et al. 2008; Liang et al. 2020; Sallam et al. 2020; Teissier et al. 2020; Villumsen et al. 2009). The training set included all F_2_ individuals and 18% of the F_3_ to F_7_ individuals. Thus, we assumed model updating. Each progeny consisted of 20 plants. The F_3_ generation consisted of 250 progeny (5000 plants). The progeny numbers from F_4_ to F_8_ were 150 (3000 plants), 150 (3000 plants), 50 (1000 plants), 20 (400 plants), and 5 (100 plants), respectively. The proportions of selected plants were 3.0, 5.0, 1.7, 2.5, and 1.2%. As reference scenarios, we also evaluated SNP-based selection using A (S,A) and A + AA (S,A + AA) values, haplotype-based selection based only on the predicted A value (H3, H6, and H9,A), selection based on the true genotypic value (GV), and no selection (NS). Under no selection, we advanced 500 individuals in each generation using the single-seed descent (SSD) method. For selection based on the true genotypic values, we used the values provided by *REALbreeding*. Under selection, owing to changes in the allele frequencies, the software does not compute genetic values. Since the genomic selection assuming model updating and high SNP density is not feasible for any self-pollinated crop, we also included a scenario with a lower cost of genotyping by using only 20% of the total number of SNPs. The average SNP density decreased to 0.1 cM. In this case, we also processed an admixture of epistasis types and assumed SNPs, haplotype sizes of 3, 6 and 9, and selection based on predicted A and A + AA. It is important to emphasize that we kept the same positions for the selected SNPs and genes and the additive, dominance, and epistatic values for the genes. We replicated each scenario 10 times.

### Modeling, software, and measures of genomic selection efficacy

We fitted the model $y=X\beta +\,{Z}_{1}a+{Z}_{2}d+{Z}_{3}{aa}+{Z}_{4}{ad}+{Z}_{5}{dd}+\varepsilon$, where $Z{\prime} s$ represent the incidence matrices associated with the vectors of A, D, AxA, additive x dominance + dominance x additive (AxD), and dominance x dominance (DxD) genetic values, respectively. The assumptions were $a \sim N(\Phi ,{\rm{A}}{\sigma }_{A}^{2})$, $d \sim N(\Phi ,{\rm{D}}{\sigma }_{D}^{2})$, ${aa} \sim N(\Phi ,{\rm{AA}}{\sigma }_{{AA}}^{2})$, ${ad} \sim N(\Phi ,{\rm{AD}}{\sigma }_{{AD}}^{2})$, ${dd} \sim N\left(\Phi ,{\rm{DD}}{\sigma }_{{DD}}^{2}\right),{\rm{and}}\varepsilon \sim N(\Phi ,{{\rm{I}}\sigma }^{2}{\rm{I}})$. Since we did not include genotype x year interaction, the vector of fixed effects includes only the population mean. The dimensions of the incidence matrices are n x n, where n is the number of individuals. The GBLUP model was implemented using the *BGLR* R package (Perez et al. 2010). We used the defaults for burn-in (500) and iterations (2000) and checked for convergence. The additive and dominance genomic matrices were computed following the methods proposed by VanRaden (2008) (first method) and Su et al. (2012b), respectively, using the *sommer* R package (Covarrubias-Pazaran, 2016). We applied the Hadamard product to the additive and dominance genomic matrices to generate epistatic genomic matrices.

We constructed haplotypes based on a fixed number of adjacent SNPs, employing our own R code. In this study, we defined haplotype sizes as 3, 6, and 9. Owing to the high time required for processing the genomic matrices with a size of 9, we processed only the admixture of types under this size. To evaluate the haplotype pattern in the F_2_ population, we used *Haploview* (Barrett et al. 2005). Haplotypes were defined using three methods. Gabriel et al. (2002) uses the 95% confidence bounds of Lewontin’s D’, the four gamete rule (Wang et al. 2002) flags evidence of historical recombination by scanning for all four possible two-marker haplotypes, and the solid spine of the LD method defines blocks based on the relative LD between the outermost SNPs and intermediate ones. Haplotyping requires phased SNP genotypes, which we conveniently obtained from *REALbreeding*’s output, where the left allele is of maternal origin. We adopted the multiallelic additive-dominance haplotype model proposed by Da (2015). Processing this model corresponds to treating each haplotype allele as an SNP, a procedure referred to as ‘pseudo-SNP’ by Karimi et al. (2018). In an analysis including **n** SNPs, assuming haplotypes of **s** SNPs, the total number of haplotype alleles (pseudo-SNPs) is (**n**/**s**) haplotypes x 2 ^**s**^ haplotype alleles. Assuming 49,823 SNPs and a haplotype size of 9, we have approximately 2,834,375 haplotype alleles.

We calculated the genetic gains by the difference between the genotypic means of successive generations (genetic gain/generation = genotypic mean of generation n + 1 minus genotypic mean of generation n; total genetic gain = genotypic mean of generation F_8_ minus genotypic mean of generation F_3_). We estimated the A value prediction accuracy by Pearson’s correlation between the predicted A values and the true genotypic values. Due to selection, *REALbreeding* does not compute the A values. We computed the genotypic variance from the true genotypic values provided by *REALbreeding*. The theoretical accuracy for phenotypic selection is the square root of the heritability (0.55). To test differences between total genetic gains, we applied regression analysis (F test) or *t* test assuming distinct variances. To compute the correlations between the number of favorable genes and the A and AxA values, we used the genotypes for genes and the true genetic values, assuming no selection from generations F_2_ to F_8_, provided by *REALbreeding*.

## Results

### F_2_ haplotype pattern and epistatic variances

**Fig. 1:**
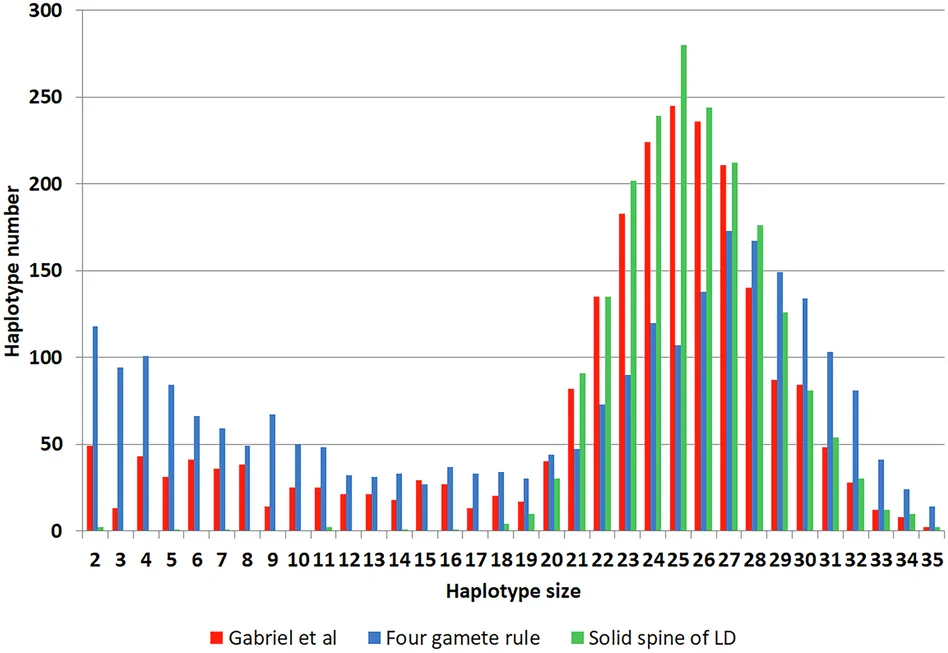
Distribution of haplotypes per haplotype size in the F_2_ generation. The haplotype methods were Gabriel et al. (2002), the four-gamete rule, and the solid spine of LD.

The analysis of the haplotype pattern in the F_2_ generation (Fig. 1) revealed, irrespective of the method, a predominance of long haplotypes (average of 22.4 SNPs). However, on average, only 4.5% of the SNPs were included in haplotypes (sizes 2 to 35). The F_2_ epistatic variance/genotypic variance ratio ranged from 1.1%, under complementary epistasis, to 13.9%, assuming duplicate epistasis. The additive variance ranged from 41.8 to 141.7% (due to negative genetic covariances) of the genotypic variance in F_2_, whereas the AxA genotypic variance ranged from 0.5 to 6.1%. In F_2_, there were some true negatives of low magnitude covariances (AxAD, AxDA, DxAA, DxAD, DxDA, DxDD, AAxAD, AAxDA, AAxDD, ADxDA, ADxDD, and DAxDD), depending on the epistasis type.

### Comparative assessment of the haplotype-based genomic selection and significance of the AxA genetic value in selection

**Fig. 2:**
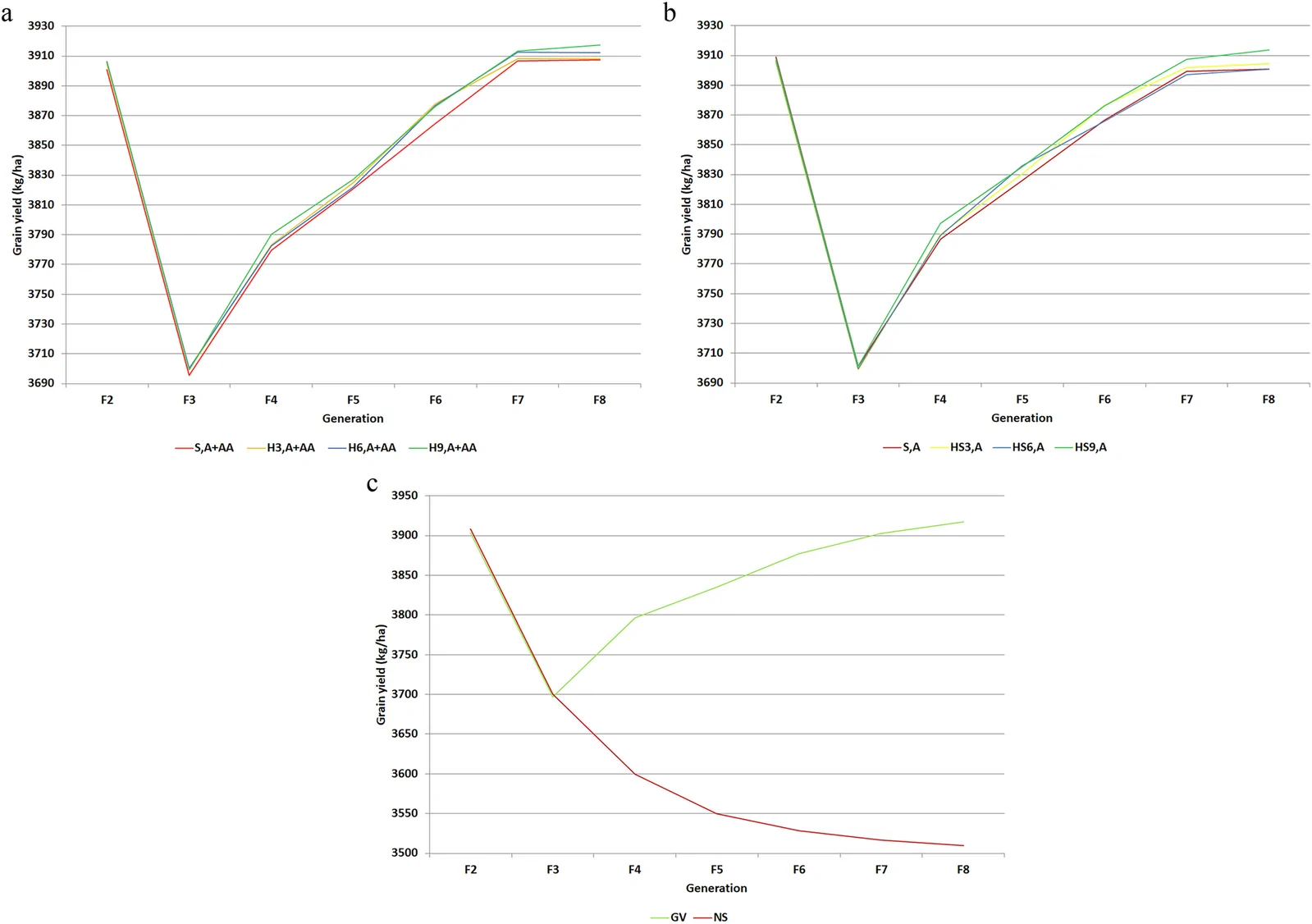
Average grain yield (kg ha^−1^) over generations for haplotype- and SNP-based genomic selection (H and S). The scenarios include haplotypes sizes 3, 6, and 9, selection using the additive plus AxA (A + AA) (**a**) and the additive (A) (**b**) values, selection based on the true genotypic value (GV), and no selection (NS) (**c**), assuming an admixture of types. The standard deviations for the means ranged from 4.0 to 36.5 kg ha^−^^1^, proportional to generation.

Under admixture of epistasis types, epistatic and AxA variances accounted for 6.4 and 2.0% of the genotypic variance, respectively. The total genetic gains from the haplotype-based genomic selection based on the predicted A + AxA values were 208.4, 212.0, and 218.1 kg ha^−^^1^ for haplotypes sizes 3, 6, and 9, respectively (Fig. 2a). However, the linear and quadratic regression analyses revealed no effect of haplotype size (R^2^ values 2.3 and 2.9%; regression P values 0.43 and 0.68, respectively). Thus, haplotype-based genomic selection resulted in an average total genetic gain of 211.4 kg ha^−^^1^. The SNP-based genomic selection using the A + AxA value provided a total genetic gain of 211.7 kg ha^−^^1^, indicating that both procedures are equivalent (P value 0.97) (Fig. 2a).

Ignoring the AxA effects on selection, the total genetic gains with haplotype-based analysis were 229.5, 204.1, and 204.1 for haplotype sizes 3, 6, and 9, respectively. Using SNP-based analysis the gain was 203.0 kg ha^−^^1^(Fig. 2b). Linear and quadratic regression analyses revealed a negative effect of haplotype size, with haplotype-based genomic selection using only the A value (R^2^ values 38.5 and 44.9%; regression *P* values 0.04 and 0.054, respectively) (Fig. 2b). Thus, the increase in the haplotype size from 3 to 9 decreased the genetic gain. Consequently, we assumed that 3 was the best haplotype size for additional comparisons of genetic gains, irrespective of the selection criterion. The difference between the genetic gains using haplotype size 3 and SNPs was statistically significant (*P* value 0.03) (Fig. 2b). A comparison of the selection criteria using haplotype size 3 revealed a statistically significant increase in selection efficacy when the predicted AxA effect on selection was ignored (*P* value 0.046). However, the difference from the SPN-based approach was not statistically significant (*P* value 0.40) (Fig. 2a, b). Selection based on the genotypic value resulted in a total genetic gain of 220.4 kg ha^−^^1^ (Fig. 2c). Compared with the haplotype-based genomic selection with a size of 3, when the A value was used as the selection criterion, the difference was not statistically significant (P value 0.55) (Fig. 2b, c). The total genetic gain with the SNP-based approach, in which A was used as the selection criterion, was statistically equal to the gain due to selection based on the genotypic value (*P* value 0.24) (Fig. 2b, c).

### Comparing epistasis types

**Fig. 3:**
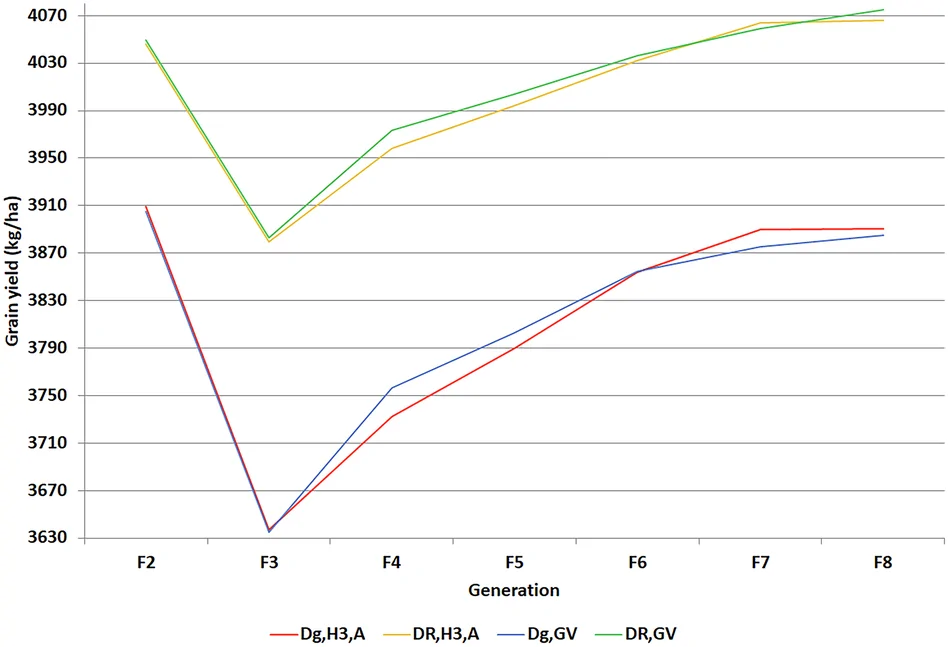
Average grain yield (kg ha^−1^) over generations for haplotype-based genomic selection (H, size 3) using the additive value (A) and selection based on the true genotypic value (GV), assuming dominant and recessive epistasis (DR) and duplicate genes with cumulative effects (Dg). The standard deviations for the means ranged from 3.8 to 46.5 kg ha^-1^, proportional to generation.

For the seven distinct epistasis types, we observed, in general, the same results: no significant haplotype size effect (P values in the range 0.19–0.94), no significant difference between haplotype- and SNP-based approaches (P values in the range 0.18–0.97), no significant effect by selection based only on the A value (P values in the range 0.08–0.99), and no significant difference between the genetic gains with genomic selection and selection based on the genotypic value (P values in the range 012–0.99) (Fig. 3). For better visualization, we present the results only for a haplotype size of 3 and selection based on A and genotypic values. Exceptions occurred for complementary and duplicate epistasis, where significant decreases in the total genetic gains occurred when the haplotype size increased from 3 to 6 (*P* values 0.04 and 0.01), but only assuming selection based on the A + AxA value. The decreases were −37.9 and −42.1 kg ha^−^^1^, respectively. Assuming non-epistatic gene interaction and selection based on the A value, we observed a positive effect of increasing the haplotype size at a *P* value of 0.06, with an increase of 29.0 kg ha^−^^1^. Under complementary epistasis and selection based on the A value, we observed, at a *P* value of 0.054, a decrease with haplotyping, relative to the SNP-based analysis, with a decrease of −33.1 kg ha^-1^. Assuming duplicate genes with cumulative effects and haplotype-based selection, we observed, at a *P* value of 0.065, a difference of 18.7 kg ha^−^^1^ between the selection criteria. Greater total genetic gain occurred with selection based on the A value.

**Fig. 4:**
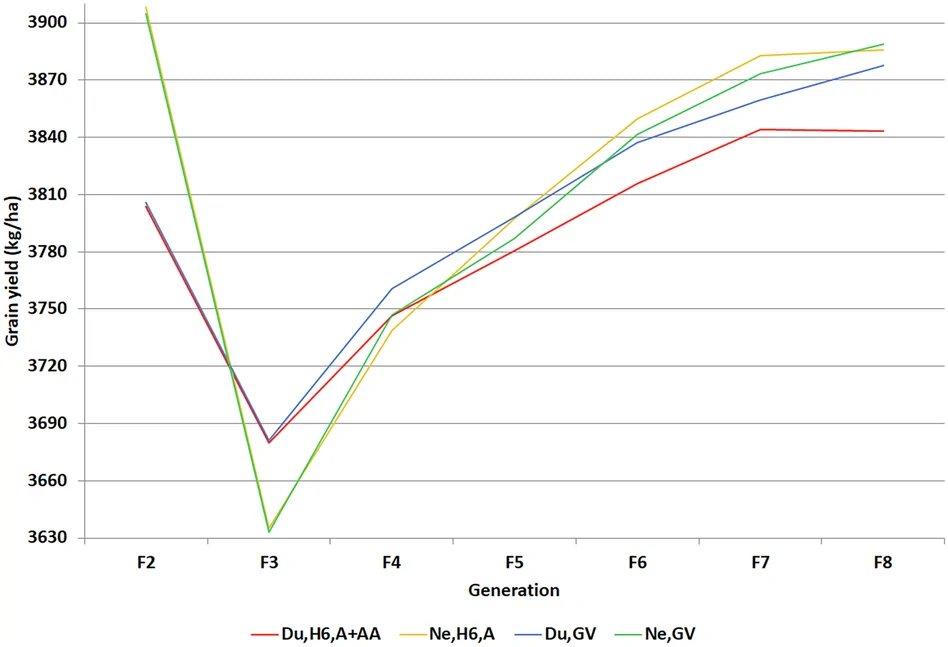
Average grain yield (kg ha^−1^) over generations for haplotype-based genomic selection (H, size 6), using the additive (A) and the additive plus AxA (A + AA) values, and selection based on the true genotypic value (GV), assuming duplicate epistasis (Du) and non-epistatic gene interaction (Ne). The standard deviations for the means ranged from 2.0 to 47.9 kg ha^−^^1^, proportional to generation.

**Fig. 5:**
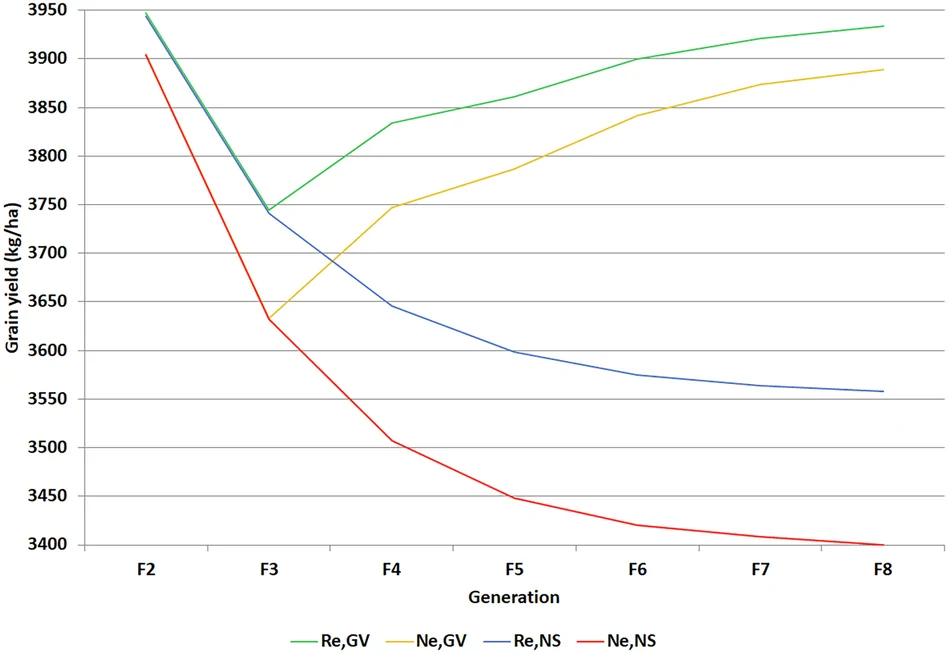
Average grain yield (kg ha^−1^) over generations for selection based on the true genotypic value (GV) and no selection, assuming recessive epistasis (Re) and non-epistatic gene interaction (Ne). The standard deviations for the means ranged from 2.0 to 46.5 kg ha^−^^1^, proportional to generation.

Duplicate epistasis minimized the total genetic gain, from genomic selection based on a haplotype size of 6 and a predicted A + AxA value (163.6 kg ha^-1^); the epistasis that maximized the total genetic gain was non-epistatic gene interaction, from selection based on a haplotype size of 6 and predicted A value (271.8 kg ha^−^^1^). The difference between these genetic gains was statistically significant (*P* value 3 × 10^−^^7^) (Fig. 4). The correlations between the ratio of epistatic variance/genotypic variance and the minimum, average, and maximum total genetic gains were negative of intermediate magnitude ( − 0.42, −0.31, and −0.23, respectively). The scenario of no selection clearly revealed how all the genomic selection methods efficiently increased the mean values of the generations (Figs. 2c and 5). In the case of no selection, due to positive dominance, the decreases in the F_8_ generation, relative to F_3_, ranged from −122.7 kg ha^−^^1^ under duplicate epistasis to −232.2 kg ha^−^^1^, assuming non-epistatic gene interaction, which was also inversely proportional to the ratio of epistatic variance/genotypic variance. With respect to the scenario of selection based on the true genotypic value, the minimum and maximum total genetic gains were 189.4 kg ha^−^^1^ under recessive epistasis and 256.0 kg ha^−^^1^, assuming non-epistatic gene interaction (Fig. 5). The total genetic gain was inversely proportional to the ratio of epistatic variance/genotypic variance (correlation of −0.24). The difference between these two gains was statistically significant (*P* value 1 × 10^−^^4^).

### Correlations between the number of favorable genes and the A and AxA values

**Fig. 6:**
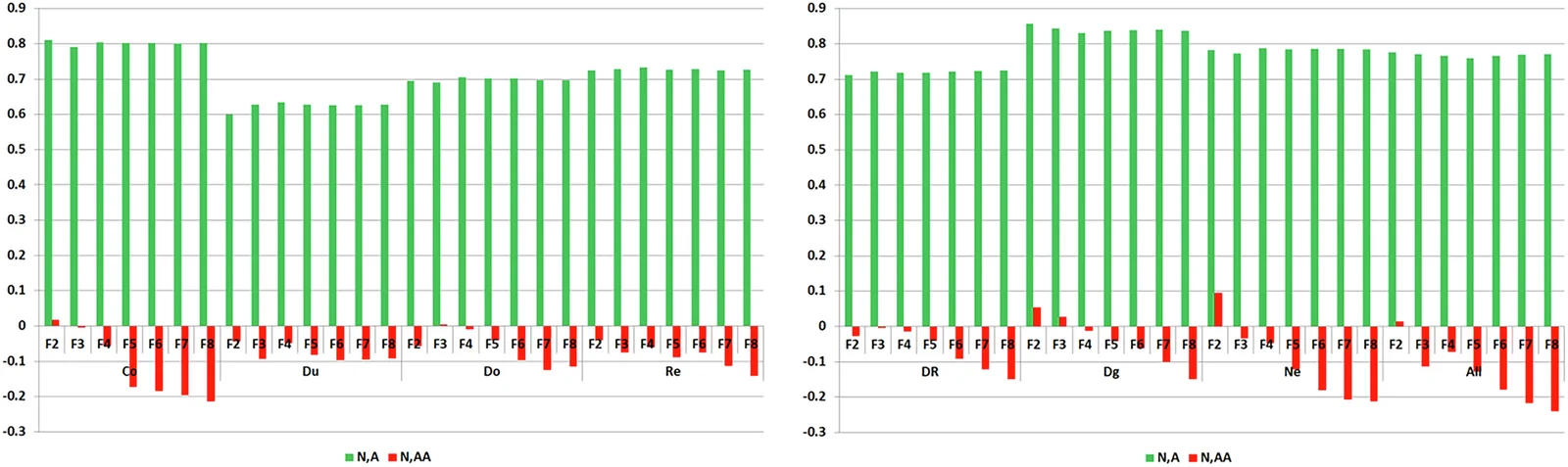
Correlations between the number of favorable genes (N) and the additive (A) and additive x additive (AA) values from F_2_ to F_8_, assuming seven epistasis and an admixture of types. Co = complementary, Du = duplicate, Do = dominant, Re = recessive, DR = dominant and recessive, Dg = duplicate genes with cumulative effects, Ne = non-epistatic gene interaction, and All = all types.

In this study, the correlations between the A and AxA values and the number of favorable genes revealed that, compared with selection based on the sum A + AA, genomic selection based on the A value was expected to be more effective due to predominantly negative values for the correlation between AxA and the number of favorable genes (Fig. 6), irrespective of the epistasis type. Based on the magnitude of the correlations, we can state that the AxA genetic values were not significantly correlated with the number of favorable genes. The correlations between the A value and the number of favorable genes ranged from approximately 0.6 under duplicate epistasis, to approximately 0.8 assuming duplicate genes with cumulative effects.

### Decrease in the genotypic variance and the prediction accuracy over generations of selection

**Fig. 7:**
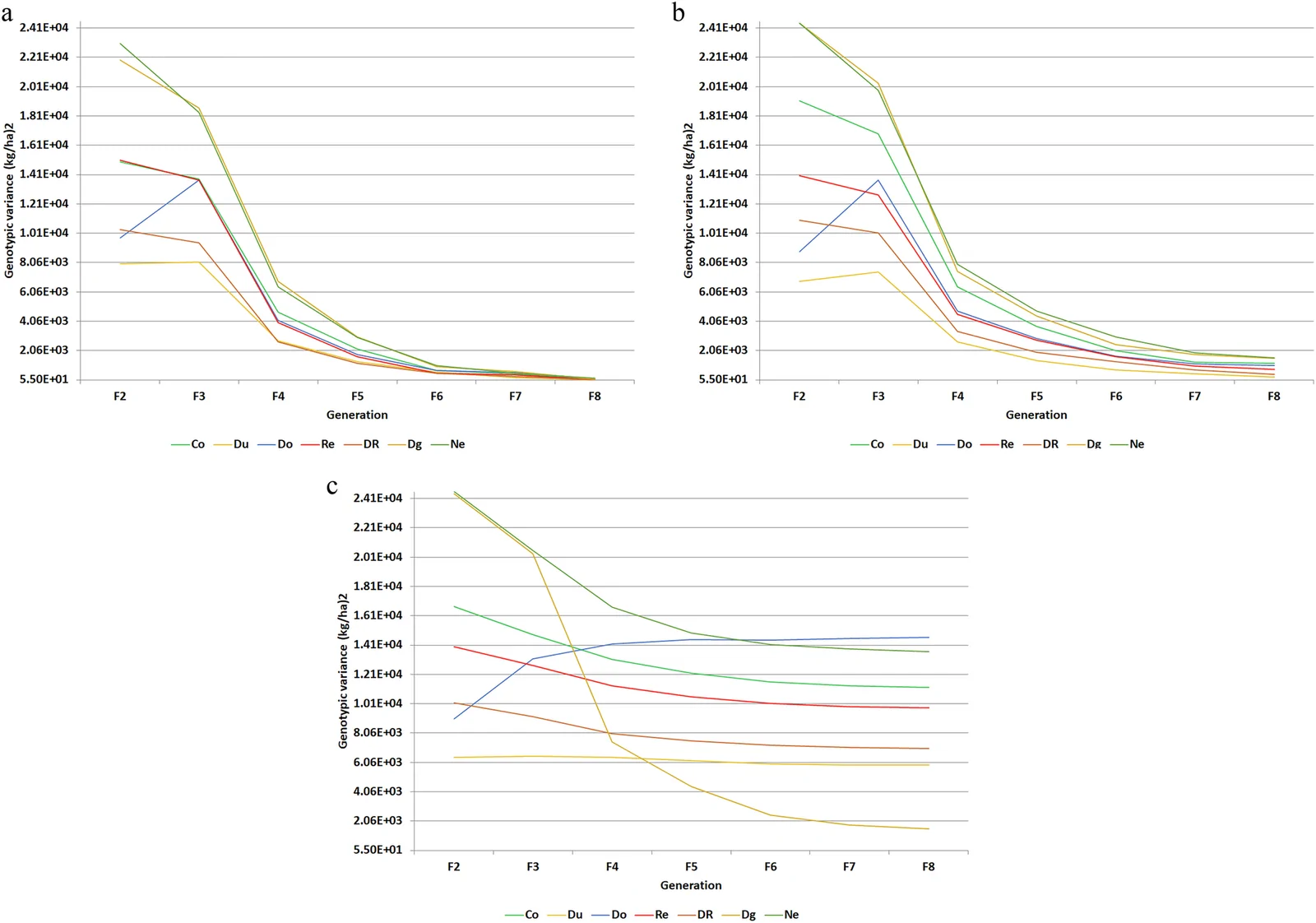
Average genotypic variance ((kg ha^−1^)^2^) for grain yield over generations. The scenarios are haplotype-based genomic selection, size 3, using the additive (A) value (**a**), selection based on the true genotypic value (GV) (**b**), and no selection (NS) (**c**). The epistasis types are complementary (Co), duplicate (Du), dominant (Do), recessive (Re), dominant and recessive (DR), duplicate genes with cumulative effects (Dg), and non-epistatic gene interaction (Ne). The standard deviations for the variances ranged from 24.3 to 4,340.0 (kg ha^−^^1^)^2^) , inversely proportional to generation.

**Fig. 8:**
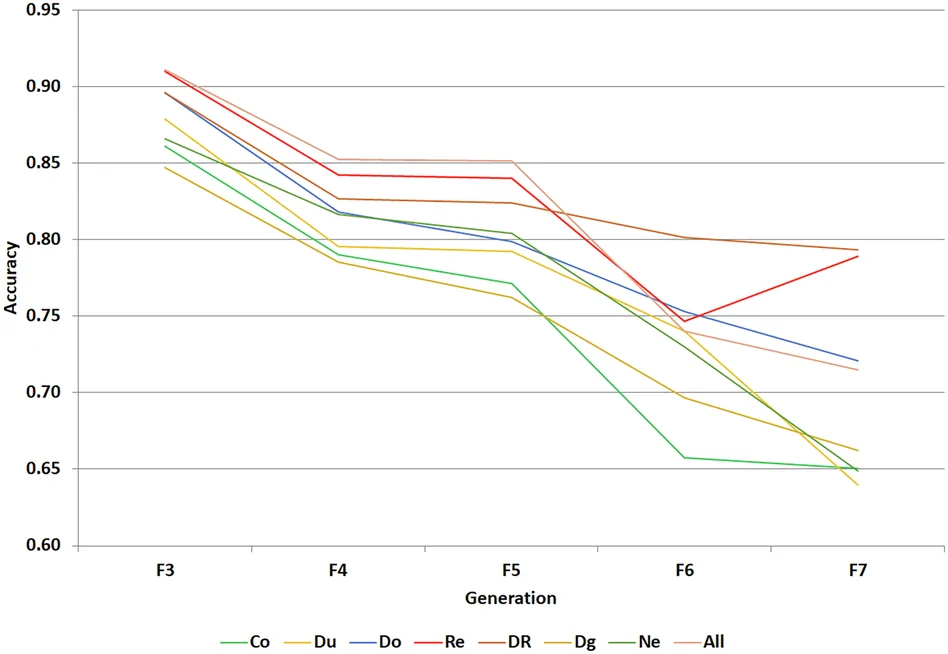
Average prediction accuracy for grain yield over generations for haplotype-based genomic selection, size 3, using the additive (A) value, assuming complementary (Co), duplicate (Du), dominant (Do), recessive (Re), and dominant and recessive (DR) epistasis, duplicate genes with cumulative effects (Dg), non-epistatic gene interaction (Ne), and an admixture of types (All). The standard deviations for the accuracies ranged from 0.00 to 0.17, proportional to generation.

As expected, all selection procedures led to a significant decrease in genotypic variance, irrespective of the epistasis type (99.0% on average) (Fig. 7a). For better visualization, we showed only the results for haplotype size 3 and selection based on the A value, for selection based on the genotypic value, and for no selection. Notably, compared with selection based on genotypic value, genomic selection led to a greater decrease (Fig. 7b). Genomic selection decreased the genotypic variances in F_8_ from 7 to 24% of the genotypic variances under selection based on the genotypic value. Additionally, under no selection, inbreeding increased the genotypic variance for dominant epistasis, but there was a decrease of variable magnitude when the other epistasis types were assumed (Fig. 7c). Excepting dominant epistasis, based on the decrease in the genotypic variance under no selection, selection was responsible for 55.0 to 92.0% of the observed decrease. Selection based on the true genotypic value led to comparable decreases, from 52.0 to 92.0%. With respect to prediction accuracy, the estimated values clearly showed how haplotype- and SNP-based genomic selection are effective in pure line breeding compared to phenotypic selection (full phenotyping; 0.55), irrespective of the epistasis type (Fig. 8). The accuracies ranged from 0.64 to 0.91, decreasing from F_3_ to F_7_. Another important result was the strong positive correlation between genetic gains and prediction accuracy. Regardless of the method and epistasis, the correlations ranged from 0.87 to 0.99.

### Impact of epistasis on the efficacy of genomic selection

Comparing the results for SNP-based genomic selection under epistasis and under no epistasis, but assuming the same genome, number and positions of genes and SNPs, parents, degree of dominance, and related factors (Silva and Viana 2025), we additionally observed that epistasis increased or decreased the total genetic gains in pure line breeding, depending on the epistasis type. The change in the total genetic gains by including and modeling epistasis ranged from −22.2 to 3.6% ( − 9.3% on average). Importantly, under epistasis all the genomic selection processes provided F_8_ progenies that were statistically equal to the superior parent (*P* values > 0.05). The differences ranged from −1.0 to 2.0%.

### Low-cost genomic selection

**Fig. 9:**
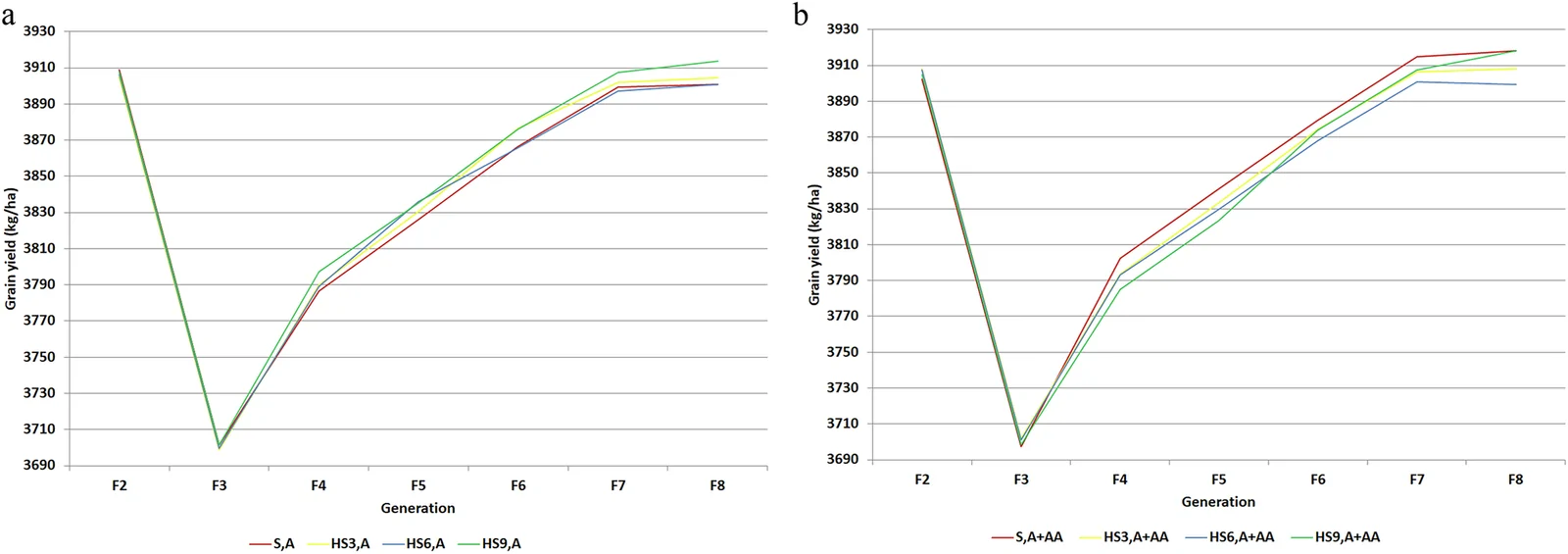
Average grain yield (kg ha^−^^1^) over generations for haplotype- and SNP-based genomic selection. The scenarios are SNPs (S) and haplotypes sizes of 3, 6, and 9 (H) using the additive plus AxA (A+AA) (**a**) and additive (A) (**b**) values, 20% of the SNPs, and admixture of epistasis types. The standard deviations for the means ranged from 4.1 to 34.5 kg ha^−^^1^, proportional to generation.

The results using 20% of the SNPs provided an impressive conclusion: genomic selection with model updating has the same efficacy under high- and low-SNP density (P value 0.5). The total genetic gains for SNPs and selection based on A and A + AA were 199.2 and 220.9 kg ha^−^^1^, respectively (Fig. 9). For haplotype size 3, we observed 205.7 and 206.6 kg ha^−^^1^. With respect to haplotype size 6, the gains were 201.0 and 198.4 kg ha^−^^1^, respectively. Using a haplotype size of 9, we observed gains of 212.3 and 219.7 kg ha^−^^1^. There were no significant differences relative to the gains observed under high density (P values ranging from 0.07 to 0.89). The decrease in the SNP density led to changes in gains ranging from −10.4 to 4.3%. Again, we did not observe haplotype size effect (*P* values for regression 0.18 and 0.17), irrespective of the selection criterion. We also did not observe significant differences between the SNP- and haplotype-based procedures (P value 0.80) or between selection based on A and selection based on A + AA (P value 0.28).

**Fig. 10:**
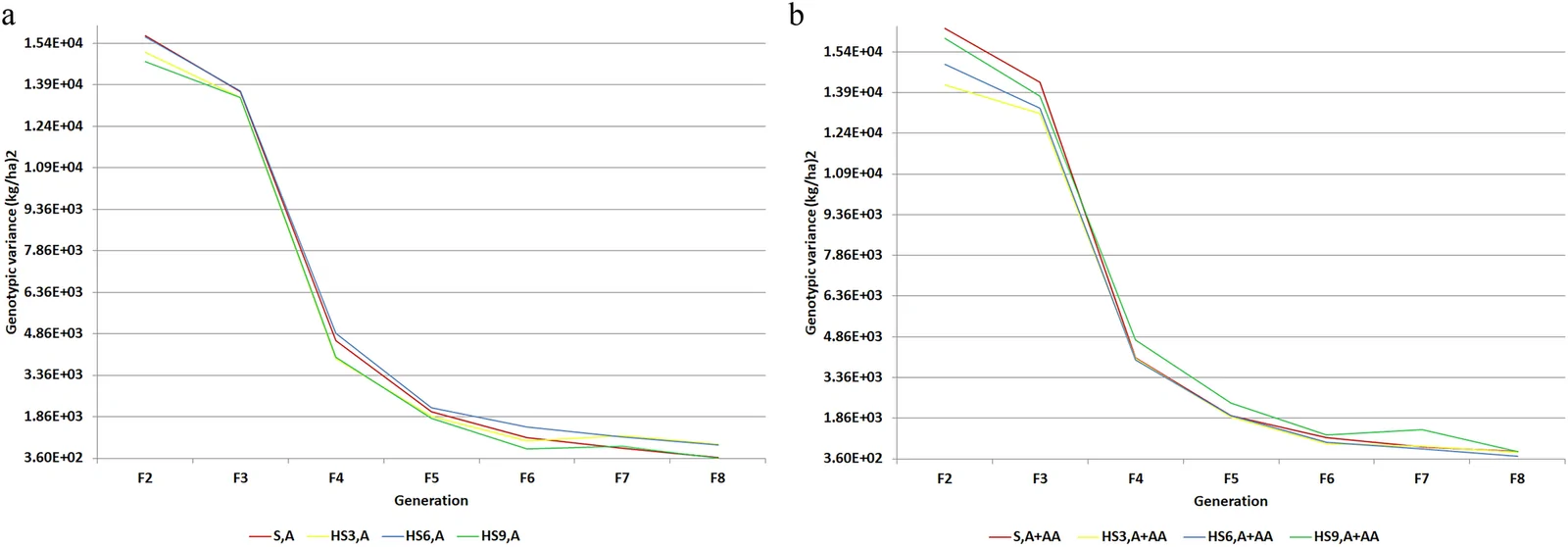
Average genotypic variance ((kg ha^−^^1^)^2^) for grain yield over generations. The scenarios are haplotype- and SNP-based genomic selection (H and S), haplotype sizes of 3, 6, and 9, using the additive plus AxA (A+AA) (**a**) and additive (A) (**b**) values, with 20% of the SNPs, and admixture of epistasis types. The standard deviations for the variances ranged from 23.6 to 1,780.7 (kg ha^−^^1^)^2^, inversely proportional to generation.

**Fig. 11:**
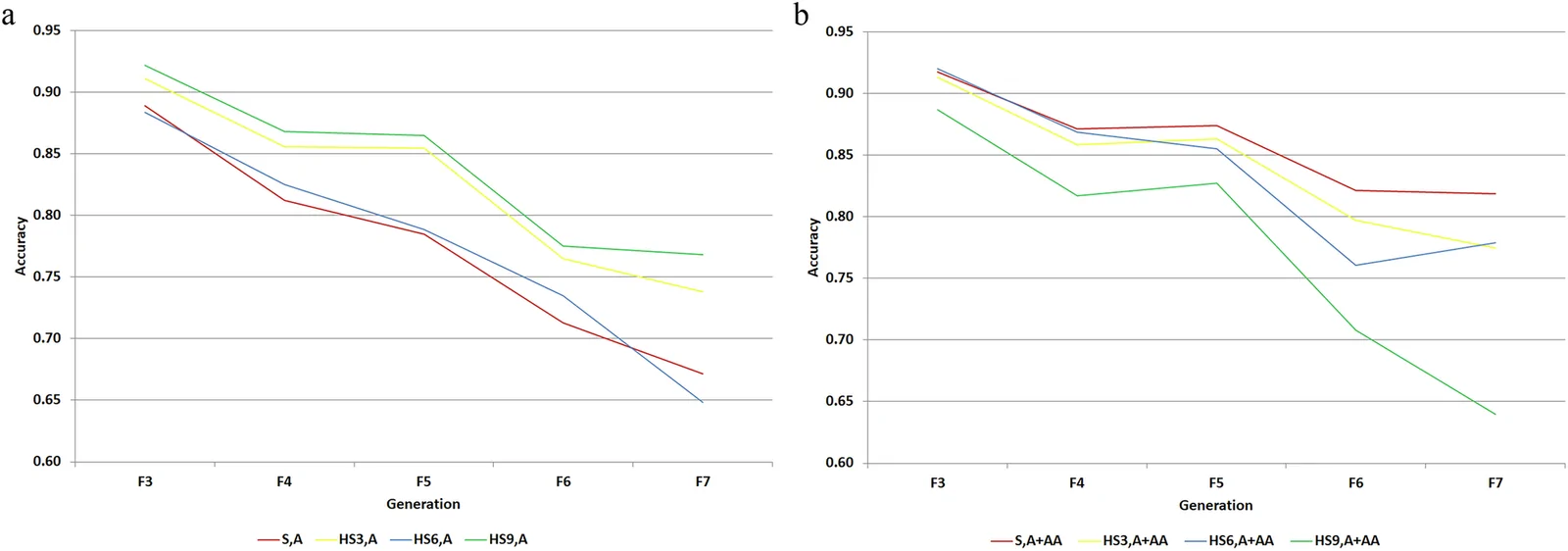
Average prediction accuracy for grain yield over generations. The scenarios are haplotype- and SNP-based genomic selection (H and S), haplotype sizes of 3, 6, and 9, using the additive plus AxA (A+AA) (**a**) and additive (A) (**b**) values, with 20% of the SNPs, and admixture of epistasis types. The standard deviations for the accuracies ranged from 0.01 to 0.19, proportional to generation.

The decreases in genotypic variability under low SNP density were very similar to the decreases observed under high density (Fig. 10). The decreases ranged from 94.2 to 97.6%. The accuracies ranged from a maximum of 0.92 in F_3_ to a minimum of 0.65 in F_7_ (Fig. 11), the same results we observed for high SNP density. All the estimated accuracies were greater than the accuracy with full phenotyping. Importantly, there were no clear differences in the standard deviations for generation means, genotypic variances, or accuracies under both SNP densities.

## Discussion

### Significance of epistasis

Based on inheritance studies for quantitative traits, using generation mean analysis, QTL mapping, and genome-wide studies (Ahsan et al. 2019), as well as transcriptome, proteome, and metabolome analyses (Samir et al. 2015), geneticists agree that epistasis is the rule and that the absence of epistasis is the exception for complex traits (Mackay 2014). Why, then, does quantifying and assessing the significance of epistasis in evolution and selection remain a challenge for geneticists and breeders, even with the help of SNPs? In general, epistatic genetic values have low significance in determining the genotypic value, relative to the A value. Additionally, the additive variance is the most significant component of the genotypic variance (Hill et al. 2008). Furthermore, epistatic effects can be difficult to quantify and falsely declared under incomplete LD at low SNP density (de los Campos et al. 2019).

### Efficacy of haplotype-based genomic selection

In this first simulation-based investigation on the efficacy of haplotype-based genomic selection under epistasis in pure line breeding, we provide clear evidence that a genomic analysis can be effective for quantifying epistatic effects, if the effects significantly contribute to determining the quantitative trait, and providing substantial genetic gains. The gains were comparable to those from selection based on the true genotypic value. When we applied haplotype-based genomic selection from F_3_ to F_7_, the total genetic gains ranged from 188.0 to 251.0 kg ha^−^^1^, depending on the epistasis type and the ratio of epistatic variance/genotypic variance. This means 38 to 50 kg ha^−^^1^ year^−^^1^. The correlation between the gain and the ratio was −0.3. Krause et al. (2023) estimated that the realized genetic gains in public soybean programs would range from 18 to 40 kg ha^−^^1^ year^−^^1^.

Although the results concerning the relative efficacy of haplotype- and SNP-based genomic selection are inconclusive, haplotyping presents expected advantages. Among them, we emphasize that haplotypes can better capture epistatic effects (Jiang et al. 2018). We showed that the heritable AxA effect can contribute to increase selection efficacy, depending on the correlation between the number of favorable genes and the AxA effect. The main reason for the nonsignificant haplotype size effect and nonsignificant difference between haplotype- and SNP-based approaches can be attributable to a reduced number of SNPs included in haplotypes in F_2_, irrespective of the haplotyping criteria. With respect to the significance of the predicted AxA effect on selection, selection based on the A values was expected to be only slightly more efficient than selection based on the A plus AxA value. The main reason for the nonsignificant effect of the AxA value on selection can be the low correlation between the predicted and true AxA values.

### Comparing selection criteria, haplotype size effects, and haplotype and SNP approaches

The two selection criteria were equally efficient. The ratio between the realized total genetic gains ranged from 0.9 to 1.2 (1.0 on average). García-Barrios et al. (2024) evaluated multienvironment prediction models and datasets for wheat and reported similar performance between a purely A model and models incorporating AxA effects. Jiang and Reif (2015) observed slightly improved prediction accuracies when AxA effects were included. Raffo et al. (2022) concluded that epistasis can increase the predictive ability, especially under conditions of high relatedness. However, the difference between the prediction accuracies was only 0.06. Weber et al. (2023) evaluated the impact of including epistatic effects in haplotype-based models and reported only marginal improvements in predictive accuracy for grain yield in wheat and for protein and oil contents in soybean, regardless of using haplotypes or SNPs.

From the assessment of the effect of haplotype size, we observed that increasing the haplotype size from 3 to 9 under an admixture of epistasis types and from 3 to 6 for the seven distinct epistasis types did not increase the efficacy of genomic selection. Unfortunately, no previous study on self-pollinated crops has assessed the effect of haplotype size based on realized genetic gains. Only on the breeding value prediction accuracy. Sallam et al. (2020) also did not observe a haplotype size effect. The prediction accuracies for haplotype sizes 5, 10, 15, and 20 oscillated from 0.31 to 0.34.

Comparing haplotype- and SNP-based approaches, we observed that both methods were equivalent, considering total realized genetic gain, A value prediction accuracy, and decrease in genotypic variance, irrespective of the epistasis type and the ratio of epistatic variance/genotypic variance. The SNP-based approach provided genetic gains from 184.0 to 247.0 kg ha^−^^1^, depending on the epistasis type and the ratio of epistatic variance/genotypic variance. Using a wheat inbred line panel, Difabachew et al. (2023) showed that predictive performance varied depending on haplotype definition and trait. Haplotypes defined by LD provided higher accuracy for disease resistance, whereas fixed-length haplotyping performed better for plant height. However, all haplotype-based approaches were less effective for grain yield. Regardless of the statistical method, inbred line panel, missing data rate, and trait, He et al. (2023) also reported equivalence in prediction accuracy for haplotypes and SNPs in rice.

### Applicability and costs of individual genomic selection with model updating

It is important to emphasize that genomic selection with model updating and phenotyping of individual plants, even for a single biparental F_2_, has prohibitive costs for genotyping and phenotyping. As breeders know, phenotyping is also expensive. This limits the applicability of high-density genomic selection with model updating and individual phenotyping in real pure line breeding programs, due to the prohibitive cost. Some alternatives for decreasing costs include the following: using a lower number of SNPs; applying genomic selection only in F_2_, combined ideally with speed breeding (Silva and Viana 2025; Viana et al. 2026); applying genomic selection based on the genotyping of parents, with a genotyping interval (F_2_ and F_5_, for example); phenotyping of progeny, using inbred progeny selection model (Viana et al. 2011); and using imputation to achieve higher density from low-density SNP panels (Niehoff et al. 2022). These alternatives can be applied for multiparent F_2_ populations in recurrent genomic selection and for several biparental F_2_ populations. Based on previous investigations comparing SNP densities (Habier et al. 2009; Solberg et al. 2008), we expected decreases in selection efficacy and prediction accuracy by decreasing the number of SNPs by 80% by random choice. However, our results revealed that genomic selection was equivalent between high (0.02 cM) and low (0.1 cM) SNP density. We observed a nonsignificant decrease of 1.3% in the genetic gains. Su et al. (2012a), who compared high (777 K) and medium (54 K) SNP densities, reported insignificant decreases in genetic gains, irrespective of the trait, statistical approach, and population. In the study of Raoul et al. (2017), genomic selection with very low density (1 K) combined with imputation provided genetic gains 15–20% lower than the gains observed from medium density (45 K), depending on the individuals genotyped. Thus, there is a threshold for the SNP density at which significant decreases can occur. In a simulation-based study in which 100 K and 54 K SNPs were processed, Zheng et al. (2022) reported an average decrease in genetic gains of only 4% with decreasing density. When 10% random, uninformative SNPs were used, Wang et al. (2024) reported changes in prediction accuracies between −0.27 and 0.11. We observed average changes ranging from −0.01 to 0.02 over generations.

## Conclusions

Regardless of the epistasis type and SNP density, genomic selection is an efficient procedure for the development of superior pure lines. Under high (a SNP each 0.02 cM) and low (a SNP each 0.1 cM) SNP density, genomic selection were equivalent. There was no effect of haplotype size on the efficacy of genomic selection. Under no selection, the AxA genetic value was negatively correlated of low magnitude with the number of favorable genes. Under selection, the inclusion of the predicted AxA value for selection did not increase the selection efficacy. Haplotype- and SNP-based genomic selection were equally efficient. Compared with traits determined only by additive and dominance gene effects, epistasis can decrease the total genetic gain in pure line breeding. Under epistasis, there was a highly positive correlation between genetic gain and prediction accuracy. There was no difference between genomic selection procedures regarding the decrease in genotypic variance.

## Data Availability

The dataset is available at https://doi.org/10.6084/m9.figshare.29825855.v1.
